# Mood States and Performance in Elite Canoe Polo Players: The Mediating Role of Stress

**DOI:** 10.3390/ijerph18094494

**Published:** 2021-04-23

**Authors:** Donatella Di Corrado, Andrea Buscemi, Paola Magnano, Nelson Mauro Maldonato, Matej Tusak, Marinella Coco

**Affiliations:** 1 Department of Sport Sciences, Kore University, Cittadella Universitaria, 94100 Enna, Italy; 2 Study Center of Italian Osteopathy and Horus Social Cooperative, 95100 Catania, Italy; andreabuscemi@virgilio.it; 3 Faculty of Human and Social Sciences, Kore University, 94100 Enna, Italy; paola.magnano@unikore.it; 4 Department of Neuroscience and Reproductive and Odontostomatological Sciences, University of Naples Federico II, 80138 Naples, Italy; nelsonmauro.maldonato@unina.it; 5 Department of Social and Humanistic Sciences in Sport, Faculty of Sport, University of Ljubljana, 1000 Ljubljana, Slovenia; matej.tusak@fsp.uni-lj.si; 6 Department of Biomedical and Biotechnological Sciences, University of Catania, 95123 Catania, Italy; marinella.coco@gmail.com

**Keywords:** team sport, psychological states, stressors, elite athletes, competition

## Abstract

Sport performance is characterized by competitive stressful conditions that elicit emotional states. The purpose of the study was to examine whether stress mediated the relationship between mood states and performance. Thirty-two elite canoe polo players from four different Italian teams (16 senior males and 16 senior females), aged between 29 and 38 years old (M = 32.3, SD = 2.71), participated in the study. Measures included level of psychological stress, six mood dimensions, and performance outcome. We also detected the digit ratio—the lower second-to-fourth digit length (2D:4D) ratio—as it was demonstrated to be correlated to high sports performance. The assessment took place one day before the first game of the national competition “ITALY CUP”. Male athletes reported lower scores on dysfunctional emotion-related states and on 2D:4D ratio than female athletes. The results of the mediation analysis showed that psychological stress plays a mediating role between moods and performance. Overall, given the limited literature, the findings supported an integrative approach to the study of the linkage between emotion and action in canoe polo.

## 1. Introduction

Canoe polo is a dynamic and contact team sport practiced on flat water or in a swimming pool, played by two teams of five players. Additionally, each team has up to three other players who can be sent in to substitute at any time. An official canoe polo match includes two 10 min game halves with a 3 min interval between periods. The goal of the game is to score into a goal, which is placed 2 m above the water. The ball in canoe polo is the same as that which is used in water polo and can be controlled by hand or by paddle. The game requires boating and ball-handling skills, excellent teamwork, and the ability to adapt to situations that change quickly and continuously. The acquisition of a high technical and physical structure as well as speed, training plans, and strategy preparation are indispensable for winning a competition. However, it must be mentioned that there are other factors to consider inherent in the competition itself, which ensure success at the highest levels of sport and might influence the results. It is extensively acknowledged that sport players must cope with a varied range of environmental difficulties and psychological responses, especially mood states, when they want to increase their sport performance [1]. Moods are temporary emotional states that individuals experience as they relate to a myriad of mutable affective states that indicate how they live through a situation at a specific instant in time [2]. A multitude of factors, such as a range of personality, environmental and contextual aspects, influence the precompetitive mood state [3]. Empirical evidence showed that people tend to experience deep psychological states when striving to achieve greatly valued goals, such as when taking part in sporting competitions [4] or academic exams [5]. Certain mood states can be recognized as one of the most important precompetitive predictive aspects of sport performance (e.g., fatigue and vigor), allowing athletes to better manage their feelings, thoughts, and actions [6]. Particularly, researchers determined that a mood—including high vigor and lower levels of fatigue, anger, stress, depression and confusion—is correlated with the best performance [7]. Stress is another factor that can negatively influence and endanger performance during competition [8]. Stress is inevitable in life as well as in sport; it is a physical, mental, or emotional request, which tends to disturb the homeostasis of the body [9,10]. Nevertheless, every athlete needs a certain stress level (such as pressure and demands) to improve one’s own game and, thus, increase one’s level of performance. Stress during sports may be acute, episodic, or chronic. Normally, it is episodic, as acute stress, if not harnessed, can affect one in the long term and damage the game [11]. Indeed, when stress is perceived as uncontainable or unmanageable, the athlete experiences a gradual to severe decrease in performance levels [12].

The relationship between mood states and performance is widely investigated [13,14,15,16,17], which also involves the deleterious effect of stress [18,19,20,21]. Therefore, not only are competitions useful as models for challenges, but they also represent profound physiological and psychological stressors. In addition to the aforementioned associations, numerous studies [22,23,24] highlight that another marker is strongly correlated with high sports performance. It is called the second-to-fourth digit length (2D:4D) ratio, with the fourth finger of the right hand relatively longer (i.e., lower 2D:4D ratio), indicating higher fetal androgen levels [22,25]. In fact, androgens are considered to be beneficial for athletic performance as they exert positive effects on muscle tissue, the immune system, and behavioral patterns; they may also contribute to the increased health status of athletes [26]. It was recently shown that 2D:4D is determined not only by prenatal androgens, but also by the balance of prenatal androgens to prenatal estrogens, which influences the development of fetal fingers [27,28]. Several studies show that male achievement in sports and athletics is correlated with a lower 2D:4D ratio [29,30,31]. Recently, associations between the 2D:4D ratio and high performance were demonstrated in female athletes for rowing [32], Olympic sports [33], and football [34]. Following these correlations, we also included this metric.

Presently, canoe polo has a growing in popularity in the sporting world. However, no scientific literature regarding the relative impact of the emotional and psychological characteristics of these athletes is available. Several studies mainly investigated the anthropometric [35], nutritional [36], hormonal [37], and physiological [38,39] requirements of male and female canoe polo players. The research highlighted that many functional alterations in both men and women players result from basic differences in body size and body composition. Nevertheless, women are highly competitive in canoe polo and have the same boat skills and technical ability as men in both slalom and sprint. Based on these arguments and given the limited research, the purpose was to investigate the mediating role of stress in the relationship between mood states and performance in canoe polo. The study presented aims to verify the role that stress plays in the linkage between performance and the six mood dimensions (tension, depression, anger, vigor, fatigue, and confusion). More specifically, we hypothesized the following:

**Hypothesis** **1** **(H1).** *There are significant relationships among stress level, mood states, and performance, in both males and females*.

**Hypothesis** **2** **(H2).** *Stress mediates the relationship between mood states and performance*.

**Hypothesis** **3** **(H3).** *Digit ratio explains a statistically significant amount of variance in stress and performance with the abovementioned moods, in both males and females*.

## 2. Materials and Methods

### 2.1. Participants

The participants in this study were thirty-two healthy and injury-free elite canoe polo players from four different Italian teams, with eight athletes each (five players on the pitch and three substitutes). The sample comprised of 16 senior males and 16 senior females, aged between 29 and 38 years old (M_age_ = 32.28 years, SD = 2.71 years). Table 1 shows the anthropometric characteristics of the participants.

The regular training schedule consisted of 2/3 h (2.3 ± 0.47) per day, distributed in eight sessions (five/six weekly workouts for the men, 5.7 ± 0.44; three/six weekly workouts for the women, 5.4 ± 1.7). Five training sessions per week were aimed for the specific technical and tactical drills (e.g., canoe polo game simulations); three sessions were devoted to non-specific physical conditioning (e.g., resistance exercise). None of the athletes received any pharmacological treatments or had any type of neuromuscular or cardiovascular disorder. Each participant signed a free written consent to participate after receiving a full explanation of the goals and the protocol of the study. All of the procedures were conducted in accordance with the ethics stated in the Declaration of Helsinki, while the University Enna Kore Internal Review Board for psychological research (UKE-IRBPSY-04.21.02) gave ethical permission.

### 2.2. Procedures

The assessment took place one day before the first game of the national competition “ITALY CUP”. Measurements were conducted individually (approximately 15 min) in a quiet room inside the training facilities, under the supervision of two researchers. All participants performed a battery of tests; confidentiality of the answers was assured. The athletes were required to answer based on their current feelings, as accurately as possible. During the assessment, no talking was permitted, and no feedback was provided. We evaluated stress and mood parameters; in addition, we characterized the second-to-fourth digit length (2D:4D) ratio. To guarantee anonymity of all participants, we labelled each team as follows:

“A” = the men’s team classified in first position;

“B” = the men’s team ranked in second position;

“C” = the women’s team classified in third position;

“D” = the women’s team ranked in fourth position.

### 2.3. Measures

#### 2.3.1. Psychological Stress

The level of stress was assessed using the Measurement of Psychological Stress [40] (MPS), a self-administered questionnaire consisting of 9 items. It is based on different aspects related to the perception that the individual has of its condition (cognitive–affective, physiological, behavioral). The response choices make use of a Likert-type scale whose possible answers are 1–4 (from ‘‘not at all’’ to ‘‘very’’). The scale maintains a test-retest stability of 0.68 to 0.80 under apparently constant conditions, with a Cronbach α coefficient of approximately 0.95.

#### 2.3.2. Mood Measurement

To gauge moods, a 30-item Profile of Mood States was used [41] (POMS). The instrument is a self-rating questionnaire consisting of six dimensions: Tension (T-state of musculoskeletal tension and worry), Depression (D-state of sadness and despondency), Anger (A-state of hostility towards others), Vigor (V-state of energy), Fatigue (F-state of tiredness), and Confusion (C-State of light-headedness). Ratings reflected their moods at the time of evaluation. Respondents rate each item on a 5-point Likert scale, with anchors ranging from “Not at all” to “Extremely.” The internal consistency values (Cronbach’s alpha) of all six dimensions were all greater than 0.90.

#### 2.3.3. 2D:4D Measurement

**Figure 1:**
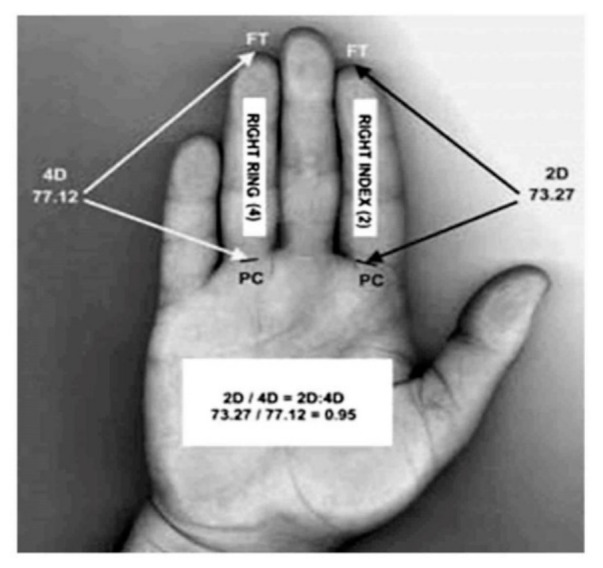
Measurement and calculation of the second to fourth digit (2D:4D) ratio.

To assess the 2D:4D ratio, each participant’s right hand was scanned on a flatbed scanner, generating images that allow for an accurate measurement of the digit length from the metacarpophalangeal crease up to the fingertip [42]. The digit ratio was calculated from right-hand measurements because right-hand digit ratios showed higher sex differences, and, thus, they are more sensitive to prenatal androgens [43,44]. Digit measurement expressed in millimeters (mm) was executed for digit two (2D) and digit four (4D) using a Vernier digital caliper 0–150 mm (Cokraft^®^, Digital caliper, Sweden) with a precision of 0.01 mm (Figure 1). The 2D:4D ratio was calculated by dividing the 2D length by the 4D length.

#### 2.3.4. Performance

Finally, performance throughout the actual match was assessed using an objective measurement derived from the final national competition outcome (teams’ ranking).

### 2.4. Statistical Analysis

Data are expressed as means ± standard deviations (*s*) and the range. A student’s t-test was used to detect the mean differences between males and females. A one-way analysis of variance (ANOVA) was performed on the scores of dependent variables (2D:4D ratio, stress, and mood states), comparing the means of each team. A Tukey’s post hoc test was used to define the existent differences. Additionally, Spearman’s rho correlation coefficient was used to determine the relationship between the selected variables. To examine the significance of mediation, the recommendations put forth by Baron and Kenny [45] were followed. Their Steps 1, 2 and 3 involve testing the significance of the relationship between the following: (1) the independent and the dependent variables (i.e., mood states and performance); (2) the independent and the mediator variables (i.e., mood states and stress); (3) the mediator and the dependent variables (i.e., stress and performance). If these steps are passed, one should determine in Step 4 whether the mediator variable reduces or eliminates the link between the independent variable and the dependent variable. For this purpose, a mediation analysis was conducted using Hayes’ [46] PROCESS version 3.1 (Hayes PROCESS macro model (Model 4)—Ohio, USA) computational tool for SPSS. This tool enables the estimation of path coefficients, standard errors, and different indexes of effect size, as well as the significance of the indirect effects obtained through the bootstrapping method with 5000 repetitions, with a confidence interval (CI) of 95% [47]. Statistical significance was set at *p* ≤ 0.05. Statistical analyses were processed using SPSS version 25.0 (IBM, Armonk, NY, USA).

## 3. Results

### 3.1. Gender Differences

Table 2 shows the comparison between males and females conducted through the Student’s *t*-test (*p* < 0.05).

As seen above, the 2D:4D ratio for the right hand was significantly lower in the male athletes than in the females. Moreover, male athletes had significantly lower levels of psychological stress and two dysfunctional mood dimensions (tension and anger). The functional mood dimension (vigor) was significantly higher in men than in women.

### 3.2. Performance Outcome, ANOVA Results, and Correlations

The final national competition outcome showed the two men’s teams (named “A” and “B”) placing first and second, respectively. Meanwhile, the two women’s teams in the competition (named “A” and “B”) finished third and fourth, respectively. The one-way analysis of variance (ANOVA) results (2D:4D ratio, stress, and mood states by team of players) were significant (Table 3). Specifically for women’s teams, a Tukey’s post hoc test showed significantly higher scores on digit ratio (C vs. B *p* = 0.007; D vs. B *p* = 0.011), stress (C vs. A *p* < 0.001; C vs. B *p* < 0.001; D vs. A *p* < 0.001; D vs. B *p* < 0.001), tension (C vs. A *p*= 0.04; C vs. B *p* = 0.02; D vs. A *p*= 0.04; D vs. B *p* = 0.03), and anger (D vs. A *p* = 0.01; D vs. B *p* = 0.04), while scores on vigor came out lower (C vs. B *p* = 0.05).

The relationships between the variables of the study were analyzed using Spearman’s rho correlation coefficient (Table 4).

As can be observed, scores for the 2D:4D ratio, stress, tension, anger, and performance variables were significantly and positively related. On the other hand, scores for the functional state (vigor) were significantly and negatively associated only with scores for stress and tension.

### 3.3. Mediation Analysis

**Figure 2:**
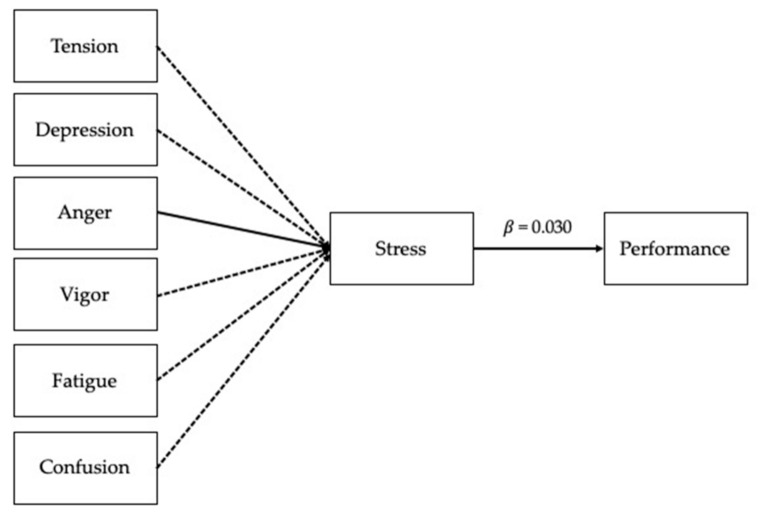
The mediation model. Note: Dashed lines indicate non-significant effects.

Based on the correlation results, a mediation analysis was executed by entering the score for stress as a mediator in the mood states and performance relationship. The mediation results are presented in Table 5, which contains the standardized β, indicating the intensity of the effect, and the 95% CIs, indicating the significance of the effect with a 5% probability of error (CIs that do not contain 0 are significant). The results showed that anger had an indirect effect, mediated by stress, on performance (IE = 0.03, CI = 0.012–0.067). Thus, the relationship between anger and performance was fully mediated by stress (partially confirming our hypothesis). Surprisingly, tension, depression, vigor, fatigue, and confusion had no direct effect on performance nor an indirect effect mediated by stress. The mediation effects are reported in Table 5 and represented in Figure 2.

## 4. Discussion

The present study aimed to evaluate the role of stress as a mediator between mood states and performance during the national competition “ITALY CUP”. We hypothesized that all the independent variables would be related to performance and the six mood dimensions (tension, depression, anger, vigor, fatigue, and confusion) through the mediation of stress. Data analysis partially confirmed the research hypotheses, highlighting that only the relationship between anger and performance was fully mediated by stress. To the best of our knowledge, there are no scientific studies describing the relative impact of emotional and psychological characteristics on canoe polo athletes. Hence, we can only compare the present results to those of similar sports.

Firstly, the comparison between males and females showed that the 2D:4D ratio, psychological stress, tension, and anger were significantly lower in the male athletes than in the female. Moreover, men had significantly higher vigor than women. Similarly, the research conducted by Brandt et al. [8], which assessed the association between mood states and the athletic performance of elite sailors in high performance competitions, women showed less vigor and greater tension, depression, anger, fatigue, and confusion. In the Segato, Brandt, Liz, Vasconcellos and Andrade [48] investigation of stress in elite male and female sailors, women had higher stress levels. Evaluating mood alterations in elite swimmers during the competition, both Raglin, Morgan and O’Connor [49] and O’Connor, Morgan, Raglin, Barksdale and Kalin [50] found significant increases in dysfunctional states (tension, depression, anger, and fatigue). Di Corrado, Agostini, Bonifazi and Perciavalle [51], found the same results, monitoring mood states and salivary cortisol during the national match in a group of female elite water polo players. In competitions with a high degree of personal importance for the players, stress can be interpreted as incontrollable; therefore, athletes may experience a disturbance in mood states, such as increased irritability and elevated tension influencing their performance [5,52,53]. In the present study, men appeared to maintain good emotional stability, specifically regarding control of stress and impulse; in fact, they were less stressed and felt more vigorous. Moreover, we found a lower 2D:4D ratio for the right hand in the male athletes than in the female, suggesting a higher prenatal androgen exposure, and confirming the association between the 2D:4D ratio and high performance.

Post hoc tests revealed significantly higher scores in women’s teams (third place and fourth place in the competition) on 2D:4D ratio, stress, tension, and anger, whereas scores on vigor were lower with respect to men’s teams (first place and second place in the competition). These findings also suggest an association between moods, a lower 2D:4D ratio, and the results achieved (e.g., the position in which the athlete finished the competition).

In addition, Spearman’s rho correlation revealed very strong and positive relationships between 2D:4D ratio, stress, tension, anger, and performance. On the other hand, scores for the functional state (vigor) were only significantly and negatively associated with scores on stress and tension. Results of the present study offer some support for the available scientific data and focus on the relationship between mood states and athletic performance [54]. Messias et al. [55] investigated the anthropometric, nutritional, genetic, psychological and sleep variables of elite slalom kayakers to verify the correlation of these variables with performance (finish time on a white-water course with 24 gates). The results showed a significant and positive relationship between fatigue and performance. Didymus, Raspin, Fletcher, and Arnold [56] investigated the stressor-coping associations experienced by elite slalom kayakers prior to a major competition. The results confirmed that the participants experienced a variety of competitive stressors in the period prior to the major competition. Alekrinskis, Bulotienė, and Dagytė [57] assessed and compared the pre-competitive emotional states of the Lithuanian national kayak and canoe rowing team members, and junior kayakers and canoeists. They found a high level of pre-competitive emotional state among members of the Lithuanian national kayak and canoe rowing team as compared to junior rowers. Ferreira, Ferreira, and Facco Stefanello [58] analyzed the association of the Brazilian Mood Scale with specific physiological variables (lactate and heart rate) in 54 Olympic kayak athletes prior to a competition. The findings showed significant differences when the highest loads were reached, thus indicating the sensitivity of the mood states associated with the intensity of the sports stimulus with respect to a state of calm and stability that shows few changes in moods.

Regarding the relative length of the second and fourth digits, our results also offer support for the notion that the 2D:4D ratio is a valid marker of sportive achievements in different disciplines, as confirmed by a number of the aforementioned studies. Di Corrado and Perciavalle [59] evaluated the relations between 2D:4D ratio and mood states in 15 high-level female water polo players, during training and the national competition. The results showed a positive relation between 2D:4D ratio and the anger mood only during the training, revealing an acquired capability of controlling the anger during the competition. Perciavalle, Di Corrado D, Perciavalle, and Coco [60] investigated the 2D:4D ratio and mood states of elite swimmers to verify the correlation of these variables with performance. The results indicated that a low 2D:4D ratio is associated (*p* < 0.01) with the best performance of male swimmers.

The last investigation of the present study—a mediation analysis—showed that only anger has an indirect effect (IE), fully mediated by stress, on performance. Surprisingly, no mediational effects of stress in the relationship between mood states and performance was found, except for anger. It was recognized that anger and tension have curvilinear effects on performance; that is, very low and very high levels of these mood states can damage performance, whereas a moderate level has a facilitating effect, as it helps to mobilize strength and attention, improving motivation, confidence, and powerful skill execution [61]. Spielberger [62] defined anger as an emotional state characterized by feelings that vary in intensity, from mild exasperation or aggravation to ferocity and rage, and it is related to the arousal of the autonomic nervous system. Most players feel excitement before the competition through physiological responses (e.g., increased heart rate and blood pressure) [63]. The more important the competition, the greater the desire; the greater the desire of the athlete to perform as well as possible, the stronger the excitement [7]. Based on these considerations, anger can disorganize and damage performance; conversely, it can energize and organize behavior towards the realization of a task [64]. Lazarus [65] demonstrated that there could be instances wherein the mobilized energy resulting from anger can improve performance, sustain effort, postpone fatigue, and maintain alertness.

The findings of the present study contribute to the current research, highlighting the mediation effect of psychological states on performance, especially during competitions [66]. Canoe polo is a physiologically and technically complex sport of interval activities. The high aerobic, anaerobic, and strength conditioning required for this needs perseverance and commitment as well as well-developed technical, tactical, and physical skills (muscular endurance, speed, coordination, and agility). Athletes who engage in competitive performances must also have a strong and balanced emotional state of mind. In the sport domain, coaches, athletes, and researchers must carefully consider these factors as inherent to competitive events in order to succeed.

Notwithstanding the promising results, a limitation of the study that should be acknowledged is that stress and mood states were assessed just once, prior to a competition. Future research should involve the assessment of these variables at the end of the competition in order to gain more accurate and extensive information, which would also establish a correlation between the sympatho-vagal activation stage and the elements related to the personality picture and the somatization of stress. Moreover, more studies investigating psychological factors in this sport are highly recommended since the vast majority of research mainly focuses on physiological responses, and these alone cannot fully explain competition outcomes.

## 5. Conclusions

The goal of each athlete is to improve the performance parameters of sport and to minimize and control interferences. Mental preparation strategies and pre-performance procedures can help athletes efficiently control their thought processes and emotional states, thus increasing the probability of achieving a successful performance. Specifically, through mental training, the competitive athlete is able to improve psychological skills (cognitive, motivational, emotional, and social); stabilize emotional responses during competition; accelerate the process of rehabilitation and recovery; improve communication processes; and develop team cohesion. Mental training can be practiced through the following techniques: goal setting, regulation of stress and activation levels, imagery and visualization techniques, mental attention and concentration techniques, and positive self-motivation. Education programs for coaches should constantly integrate psychological preparation as a fundamental element of an athlete’s good performance.

## Figures and Tables

**Table 1:** Canoe polo athletes’ anthropometric characteristics. Mean ± s (range).

Sex	Age	Training Time	Weight (kg)	Height (cm)
Male	33.2 ± 2.4 (29–38)	8.1 ± 3.9 (4–14)	78.2 ± 5.3 (70–92)	177.2 ± 3.6 (170–182)
Female	31.4 ± 2.7 (29–36)	7.3 ± 2.9 (4–12)	62.4 ± 5.3 (51–74)	168.7 ± 4.3 (160–175)

**Table 2:** Gender differences in the dimensions of the study. (Student’s *t*-test, *p* < 0.05).

Dimensions	Male		Female		*t*	*p*
	M	SD	M	SD		
Digit Ratio	0.92	0.02	0.96	0.03	−3.90	0.001
Stress	59.38	6.15	95.00	16.18	−8.23	0.000
Mood States						
Tension	42.00	2.63	50.19	7.13	−4.30	0.000
Depression	43.19	3.90	46.31	7.13	−1.53	0.138
Anger	45.25	3.97	54.63	11.62	−3.05	0.007
Vigor	62.63	5.41	56.56	6.70	2.81	0.009
Fatigue	45.94	8.46	50.94	11.07	−1.43	0.162
Confusion	43.44	8.71	47.81	12.15	−1.17	0.252

**Table 3:** Analysis of Variance (ANOVA) Results.

Variable and Teams	M	SD	F	η*_p_*^2^
2D:4D ratio				
A	0.93	0.02	5.76 ^†^	0.38
B	0.92	0.01		
C	0.96	0.03		
D	0.96	0.03		
Stress				
A	59.88	6.17		
B	58.88	6.51	21.87 ^†^	0.70
C	92.38	15.63		
D	97.63	17.34		
Tension				
A	42.25	3.53		
B	41.75	1.48	5.78 ^†^	0.38
C	50.25	6.90		
D	50.13	7.84		
Anger				
A	43.50	3.62		
B	47.00	3.70	4.92 ^†^	0.35
C	50.50	11.50		
D	58.75	10.87		
Vigor				
A	61.75	5.80		
B	63.50	5.23	3.03 *	0.25
C	55.00	4.60		
D	58.13	8.34		

* *p* < 0.05; ^†^ *p* < 0.001.

**Table 4:** Correlation coefficients (Spearman’s rho).

Variable	1	2	3	4	5	6	7	8	9
1. 2D:4D	1								
2. Stress	0.532 ^†^	1							
3. Tension	0.654 ^†^	0.788 ^†^	1						
4. Depression	0.243	0.365 *	0.493 ^†^	1					
5. Anger	0.215	0.551 ^†^	0.551 ^†^	0.520 ^†^	1				
6. Vigor	−0.234	−0.417 *	−0.413 *	−0.294	−0.070	1			
7. Fatigue	0.235	0.386 *	0.457 ^†^	0.677 ^†^	0.213	−0.397 *	1		
8. Confusion	0.188	0.339	0.561 ^†^	0.737 ^†^	0.477 ^†^	−0.418 *	0.642 ^†^	1	
9. Performance	0.452 ^†^	0.788 ^†^	0.600 ^†^	0.230	0.569 ^†^	−0.252	0.108	0.040	1

*p < 0.05; ^†^ *p* < 0.01.

**Table 5:** Effects of mood states on performance through stress (standardized β).

Paths	Indirect Effect		Direct Effect		Total Effect	
	β	CI. 95%	β	CI. 95%	β	CI. 95%
T–Stress–Performance	0.055	0.019–0.144	−0.031	−0.127–0.035	0.024	−0.062–0.100
D–Stress–Performance	0.018	−0.065–0.109	0.035	−0.077–0.182	0.052	−0.068–0.194
A–Stress–Performance	0.030	0.012–0.067	0.043	−0.011–0.102	0.073	0.04–0.135
V–Stress–Performance	−0.022	−0.077–0.004	−0.043	−0.095–0.010	−0.065	−0.128–−0.016
F–Stress–Performance	0.001	−0.020–0.049	−0.006	−0.090–0.028	−0.004	−0.057–0.038
C–Stress–Performance	−0.020	−0.065–0.013	−0.053	−0.092–0.008	−0.072	−0.121–−0.006

## Data Availability

The data presented in this study are available in Supplementary Materials.

## Supplementary Materials

The following are available online at https://www.mdpi.com/article/10.3390/ijerph18094494/s1.
